# Kinase independent oncogenic cyclin D1

**DOI:** 10.18632/aging.100773

**Published:** 2015-07-07

**Authors:** Mathew C. Casimiro, Andrew Arnold, Richard G. Pestell

**Affiliations:** Thomas Jefferson University, Philadelphia, PA 19107, USA

Strong evidence implicates cyclin D1 overexpression as a driving force in breast cancer and many other types of human tumors. Cyclin D1 overexpression is found in up to 50% of human breast cancers, and the pattern of cyclin D1 overexpression in tissues along the spectrum from normal epithelium to invasive breast cancer suggests its involvement in the earliest stages of mammary carcinogenesis. The importance of *cyclin D*1 as a driver oncogene is reinforced by the frequent clonal selection of *cyclin D*1 gene amplification, found in 15-20% of breast cancers, associated with poor prognosis, and by the fact that tissue-specific overexpression of cyclin D1 in transgenic mice results in mammary hyperplasia and adenocarcinoma [1]. However, the precise mechanisms through which cyclin D1 overexpression contributes to breast tumorigenesis have been controversial. Specifically, while cyclin D1's role in the pathogenesis of breast cancer may well involve, at least in part, the well-established binding/activation of its catalytic partner kinases CDK4/6, with subsequent hyperphosphorylation of pRB and G1-S cell cycle transition, several lines of evidence have suggested that cyclin D1, especially when overexpressed in the setting of cancer, may also act through other, CDK-independent, mechanisms. These alternative mechanisms of cyclin D1 action carry tremendous potential significance, for example in the rational targeting of new therapeutic agents. Our recent study [2] is perhaps the most direct test of this hypothesis performed in a highly relevant in vivo model system.

The induction of chromosomal instability is known to promote genetic rearrangements, tumorigenesis and the molecular genetic chaos associated with poor outcome cancers. The early drivers to chromosomal instability are poorly understood. Our recent studies showed that modest overexpression of cyclin D1 is sufficient for the induction of chromosomal instability within 3 cell divisions, both *in vitro* and *in vivo*. Furthermore we showed the induction of CIN occurred independently of the kinase function. In ChIP-Seq cyclin D1 associates with genes governing chromosomal instability (CIN) [3]. Using a kinase dead mutant of cyclin D1 (cyclin D1^KE^) we showed cyclin D1 induced - mitotic spindle architecture changes of chromosomal instability and supernumerary centrosomes aneuploidy and other features of CIN. In cdk4/6^−/−^ 3T3 cells cyclin D1^WT^ and cyclin D1^KE^ induced aneuploidy to a similar degree compared to control cells. Cyclin D1^KE^ induced aneuploidy to a similar extent in absence or presence of cdk4/6 agonist. Crucially, sustained transgenic expression of cyclin D1^KE^ induced mammary adenocarcinoma with similar kinetics to that of a cyclin D1^WT^ transgene [1]. ChIP-Seq studies demonstrated recruitment in the context of local chromatin of either cyclin D1^KE^ or cyclin D1^WT^ to the genes governing CIN. Thus, cdk-activating function of cyclin D1 was not necessary for the induction of either chromosomal instability or murine mammary tumorigenesis.

Understanding the different contexts and causes of cyclin D1 overexpression in breast cancer may be exceedingly important in considering its tumorigenic mechanisms. In this regard cyclin D1 knockout mice are resistant to breast cancer, however recent studies have shown that cyclin D1 genetic deletion abrogates the formation of progenitor cells that in turn give rise to cancer. *Cyclin D1^−/−KE rescue^* mice are resistant to ErbB2 mediated tumorigenesis [4]. Elegant studies by Hinds' group identified a progenitor population of cells in mouse mammary gland (parity-identified mammary cells: PI-MEC) that require cyclin D1 kinase activity for self renewal and differentiation [5]. An analysis on the *cyclin D1^−/− KE rescue^* confirmed that the resistance to ErbB2 driven tumorigenesis is linked to near total absence of the PI-MEC, making those progenitor cells the likely target for ErbB2 induced tumorigenesis. Cyclin D1 kinase activity is therefore required for mammary progenitor cells self-renewal and activity, highlighting this key role for cyclin D1 during development. In contrast, our recent study of mice bearing the MMTV-*cyclin D1^KE^* transgene addressed whether cyclin D1 overexpression can directly induce mammary tumorigenesis in a kinase independent manner, with a robust answer of ‘yes’.

The MMTV-*ErbB2* and MMTV-*cyclin D1* models are distinct. MMTV-*ErbB2* induced mouse tumorigenesis represents a good model for human breast cancers with *HER2* amplification, and the role of cyclinD1 when expressed secondary/downstream to other events (like *ErbB2* amplification) may well be kinase dependent. In contrast, MMTV-*cyclin D1* mice better represent the sizeable group of *cyclin D1*-amplified cancers, induced by primary, driver-level overexpression of the *cyclin D1* oncogene. *Cyclin D1* oncogene activation is a prevalent molecular driver of human cancer, playing key roles in breast, squamous cell, esophageal carcinoma, mantle cell lymphoma, multiple myeloma, and many other devastating human malignancies. Our novel *in vivo* evidence shows this mechanism can be kinase-independent, counter to the generally accepted “CDK-centric” paradigm of the tumorigenic activity of cyclin D1. In the clinical realm, these observations suggest that some therapeutic agents now approved or in development, most notably CDK4/6 inhibitors, will have limited efficacy in *cyclin D1*-amplified cancers since they target only the kinase partners of cyclin D1. Thus, without minimizing the improved progression-free survival associated with pharmacologic CDK4/6 inhibition in ER+ breast cancers, our data suggest that in the subset of tumors with, and potentially addicted to, *cyclin D1* amplification or rearrangement, a more effective path to impactful tumor shrinkage as opposed to slowing of growth will be to develop agents that target cyclin D1 directly. In summary, while cyclin D1 kinase activity is important in tumorigenesis, additional distinct kinase-independent mechanisms, including induction of chromosomal instability, are helping drive some tumors and attempts to exploit this finding using precision medicine should be encouraged.
